# Wild bees respond differently to sampling traps with vanes of different colors and light reflectivity in a livestock pasture ecosystem

**DOI:** 10.1038/s41598-022-10286-w

**Published:** 2022-06-13

**Authors:** Roshani S. Acharya, Joan M. Burke, Timothy Leslie, Kelly Loftin, Neelendra K. Joshi

**Affiliations:** 1 Department of Entomology and Plant Pathology, University of Arkansas, Fayetteville, AR 72701 USA; 2 USDA-Agricultural Research Service, Booneville, AR 72927 USA; 3 Department of Biology, Long Island University, Brooklyn, NY 11201 USA

**Keywords:** Ecology, Behavioural ecology, Biodiversity, Population dynamics

## Abstract

Wild bees are important pollinators and monitoring their abundance and diversity is necessary to develop conservation protocols. It is imperative to understand differences in sampling efficiency among different trap types to help guide monitoring efforts. This study used a new vane trap design to collect bees in a livestock pasture ecosystem and examined the impact of six different vane colors on wild bee sampling. We recorded 2230 bees comprising 49 species and five families. The most abundant species were *Augochlorella aurata* (25.8%), *Lasioglossum disparile* (18.3%), *Lasioglossum imitatum* (10.85%), *Agapostemon texanus* (10.8%), *Melissodes vernoniae* (9.9%) and *Halictus ligatus* (4.7%). Traps with bright blue vanes captured the greatest number and diversity of bees as compared to traps with bright yellow, dark blue, dark yellow, and purple vanes. Red vanes had the lowest captures rates of individuals and species. Different colors were associated with different bee species arrays and only nine species were found in all vane color types. Vanes with higher light reflectance properties (within 400–600 nm range) attracted the greatest number of bees. These results show that different light wavelengths and reflectivity of vane traps influence bee capture rates, and such findings can help optimize bee sampling methods in different ecosystems.

## Introduction

Monitoring the status of pollinators in agricultural farmland and other habitats is important because more than 75% of flowering plants including 35% of crops benefit from insect pollinators^1,2^. While special focus has been given regarding the distribution, diversity, and abundance of bees in agricultural farm land^1^, non-bee insects such as flies, wasps, beetles, and butterflies are also regarded as valuable pollinators but may not be as efficient as bees^3^. In order to regularly assess the populations of pollinators, simple, unbiased, and reliable sampling methods^4^ are needed.

Active and passive sampling methods are widely used to assess pollinator populations and communities in different habitats. Sweeping, hand-netting, and vacuuming are some of the active sampling methods^5^ that are generally time consuming, biased, require skilled manpower and experience of survey^6^. In contrast, passive sampling methods are considered easier as they do not require skilled manpower, and allows collection of data within a specified time range^6^. The most common passive sampling methods used for monitoring insect pollinators are Malaise traps, pan traps and vane traps^7,8^.

Among passive traps, pan traps have widely been used and successfully adopted for sampling pollinators^9^. However, capture of insects in pan traps varies with habitats^9^ and they are often less effective when used for sampling insects such as butterflies and moths^10^. Colored sticky traps have been successfully used to sample arthropods, but these traps vary in their ability to trap hymenopterans and coleopterans^11,12^. Other passive traps such as colored malaise trap are also used but are not very effective for sampling different pollinators^8^. Recently, vane traps have shown great potential for sampling bees as well as other pollinators^13^. These traps are commercially available in blue and yellow colors that act as visual cues to attract bees and other insects. In previous studies, blue vane traps had been found effective in sampling a diverse array of wild bees compared to yellow vane traps and different colored pan traps^13,14^. However, there is no information on how changing the color and design of these vanes affect overall sampling.

Visual cues help bees and other pollinators to discover the floral resources in their foraging landscapes. Bees also use their olfactory signals if the source is ~ 30 cm away and visual cues when they are closer to flowers^15^. Color vision of bumblebees and honey bees is important because it impacts their ability to detect resources^16^. Bees are known to visit naturally preferred flower color during their first flower visit and from that experience they recognize the rewarding flowers with its colors^17^. Based on the information on bee vision, researchers have used different colors in traps used for sampling bee species. Commonly used colors in passive traps are white, yellow, and blue^6,10^. Light reflected from different color traps have different intensity and thus affects number as well as species of bees and other insects that are attracted toward the trap^10,14,18^.

Vane traps, which consist of a collection jar attached to a set of colored vane panels, are an effective method for capturing a wide variety of bees found in both open landscapes and woodlands^13^ and have recently been used to sample wild bee populations in agricultural crops^19^. Vane traps provide a simple and effective sampling method to assess relative abundance of pollinators in a large area during the entire season^7^. It is considered one of the best methods to study native bee populations in grasslands^20^ and other similar habitats. However, the choice of vane colors in currently available traps is limited to blue and yellow. Therefore, there is a need to refine these traps by optimizing vane colors and design and by assessing their effectiveness for sampling bees in different ecosystems, including livestock pastures. In this context, we tested the research hypotheses that changing the color and other characteristics of vanes affect bee species capture rate in passive traps. Vanes with different colors (viz. dark blue, bright blue, dark yellow, bright yellow, purple, and red) were designed and evaluated for their light reflectance properties and attractiveness to bees in livestock pasture.

## Methods

### Study site description

The research was conducted during the summer of 2017 at the USDA-ARS Dale Bumpers Small Farms Research Center in Booneville, Arkansas (35.09°N, 93.95°W). Soil in the research site location was Leadvale silt loam, characterized by moderately well drained, nearly level or with gentle sloped landscape (https://websoilsurvey.sc.egov.usda.gov). Enders silt loam (clay mixed) was predominant in higher elevation whereas Leadvale silt loam (fine silty and siliceous) type of soil was found in lower elevations ^21^. Total annual rainfall during 2017 was 944.0 mm with highest rainfall during May (145.5 mm) and lowest during February (78.2 mm). Average annual temperature during same time was 17.6 °C with highest during July (24 °C) and lowest during January (4 °C).

### Field preparation

A managed field site (1.6 ha) was used in which mixed native grass, forbs and legumes were established in a livestock pasture. Prior to establishment of pasture, the field was sprayed with glyphosate (41% as Roundup, 0.764 L ha-1; Ragan and Massey, Inc., Ponchatoula, LA) during June, July, September, October of 2016 and January 2017, and with 75% Sulfosulfuron (Outrider; Monsanto, St. Louis, MO; 0.016 L ha-1) during September 2016 using Continental Belton cluster nozzle sprayer (Continental Belton McAlester, SR:A44117, Oklahoma city, OK). The field was burned in September 2016 and seed bed prepared using tiller (Maschio Gaspardo North America Inc., SC 300, Des Moines, IA) and rolled using 12’ Big Guy Roller (Grahl Manufacturing, St. Louis, MO) on October 2016. Topsoil at 0 to 15 cm was tested for soil fertility before seeding at University of Arkansas soil test lab which determined that no supplemental fertilizer was required during the entire study period (https://aaes.uark.edu/technical-services/soil-testing-and-research-laboratory/). The seed-mixes were Buck’s Hangout (Hamilton Native outpost, Elk creek, MO; www.hamiltonnativeoutpost.com;14.5 kg ha-1), Tallgrass Inexpensive mix and Tallgrass Exposed Clay subsoil mix (Prairie Moon, Winona, MN; www.prairiemoon.com; 13.44 kg ha-1 and 26.8 kg ha-1, respectively according to nursery recommendations), planted in February 2017. Species percentage per seed mixes and recommended rate are available in the websites of seed suppliers and are also presented in the supplementary table (S1).

### Design of sampling trap and wild bee sampling procedure

**Figure 1:**
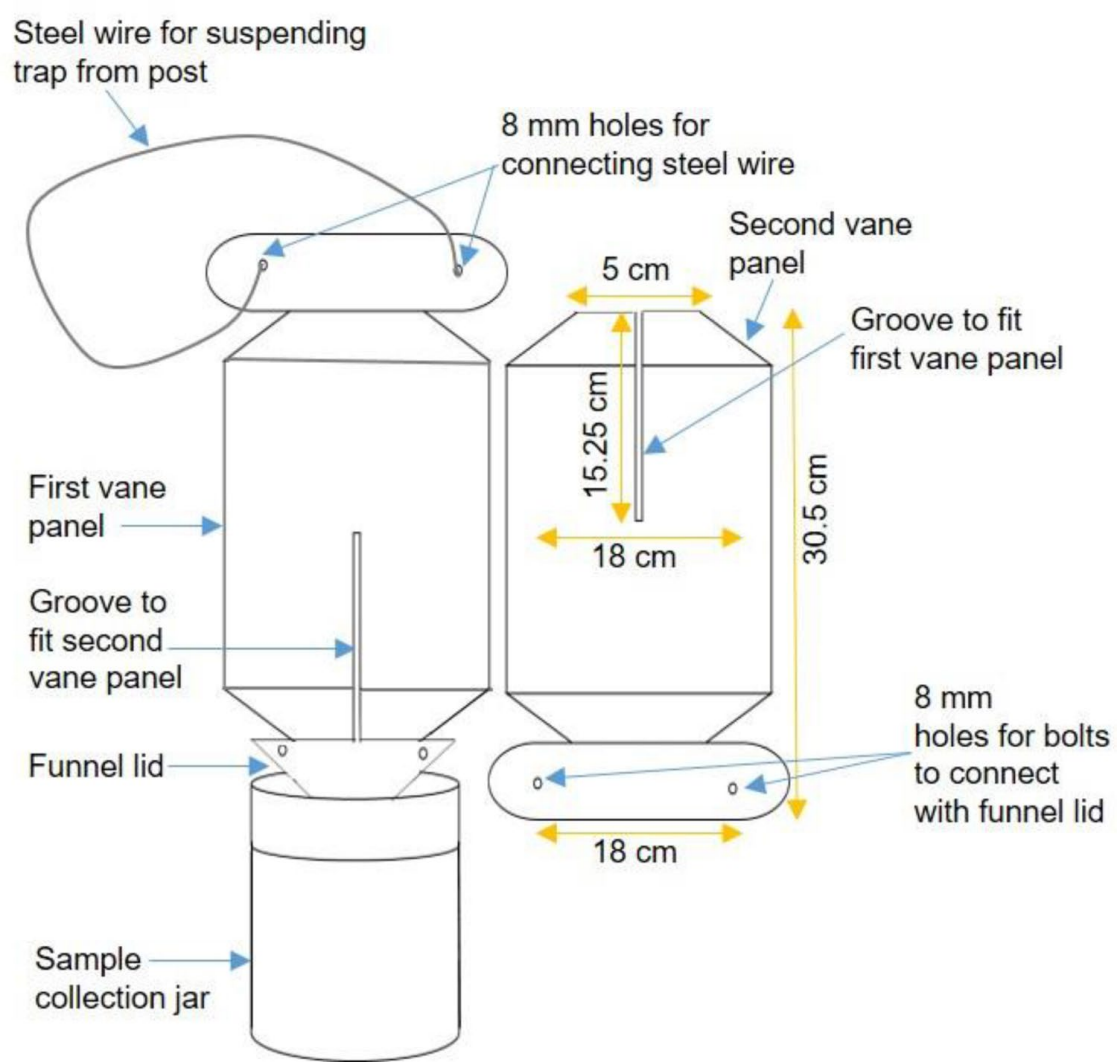
Design of trap used for sampling of wild bees in livestock pasture system. Design and illustration by N. Joshi. Specific information related to trap parts and color is given in the methods section.

Vane traps were designed in the laboratory by selecting different colors of vane panels (Fig. 1). In each trap, two panels of vanes of equal size were fitted together with panel grooves in opposite direction (Fig. 1). Panel grooves were also glued together and then fitted perpendicularly into a funnel shaped lid with the help of two small steel bolts. A single panel of vane was 30.5 cm high and 18 cm wide (in the center). The base of each panel was trimmed by bending the remainder of panel to fit with the funnel-lid by steel bolts. The top of one of the panels was as wide as the center portion but contained two small holes (8 mm, one in each half of the panel) that were used to connect to a steel wire to suspend the traps from post (Fig. 1), and the top of other panel was trimmed to fit the design and was 5 cm wide. Vane panels were made of plastic sheet of different colors (dark blue, dark yellow, purple; Interstate Plastics, Sacramento, CA). The plastic vane panels of additional traps were covered by a micro prismatic sheeting reflective tape (bright blue, bright yellow, and red; TapeCase Ltd., Elk Grove Village, IL). The color of vanes and other parts of these traps were similar in their hue to corresponding RAL color codes as follows: vane colors -bright blue: RAL5002, dark blue: RAL5011, purple: RAL4001, bright red: RAL3020, dark yellow: RAL1006, bright yellow: RAL1023; funnel: RAL5014; jar: RAL6021; wire: RAL9006; bolt: RAL9018 and post: RAL6009. The vanes with their funnel lids were then connected to a semi-transparent plastic jar (950 ml) for sample collection^14^. The same type of collection jar was used in all colors of vane traps.

Four plots each of 0.4 ha were used for the study. In each plot, six different colored vane traps (dark blue, bright blue, dark yellow, bright yellow, purple, and red) were deployed in randomized order in four linear transects (one per plot). These traps were suspended using metallic wire from the post about 1.5 m above the ground and were placed 25 m apart. The border distance from outer post was 8 m, and adjacent area was under pasture production. Traps were deployed continuously during the entire study period (June 21—August 11), and insect samples were collected twice every week, 48 h after filling with soapy water. Insects were collected directly in vials containing 70% ethyl alcohol as a preservative.

Bee samples were processed and curated in the lab and were identified to the lowest taxa. Samples were identified by Drs. D. Biddinger (Penn State University, PA), and R. Jean (Environmental Solution & Innovations, Inc., IN) using dichotomous keys^22–24^ and other available online taxonomic resources, such as different guides for species identification available in Discover Life (www.discoverlife.org )^24^.

### Light reflectance analysis of vanes of sampling traps

Light reflectance characteristics of all types of colored vane panels and sample collection jars used in passive traps were analyzed at the Department of Chemistry and Biochemistry, University of Arkansas, Fayetteville USA using a similar procedure as previously described^18^. A small square piece (~ 2 cm^2^) of each color vane was cut and kept inside the incident light window of spectrophotometer where light was reflected from the vane sample on the detector for 200 s to record reflectance. Each piece of plastic was placed in front of the barium sulfate coated detector. Total reflectance of different colored vane traps was measured within 190–1600 nm range using spectrophotometer (JASCO V-780, JASCO Corporation, Easton, MD, USA). Spectrometer containing spectroscopy software related to Windows 7 pro (64-bit) operating system was used for measuring intensity of light passing through sample.

### Data analyses

Bee abundance, species richness, similarity, and community assemblage patterns were analyzed and compared among all six colors of vane traps. Effect of vane color on bee abundance was examined by conducting one-way repeated measures analysis of variance (ANOVA)in R software program (R version 3.6.2). In addition, a Bonferroni corrected post hoc comparison test was conducted to detect significant differences among all six-vane colors. Datasets were square-root transformed to address non-normality (right-skewness) in the original dataset by using the Shapiro–Wilk normality test.

Bee species richness was compared among vane colors by developing sample-based and individual- based rarefaction curves in EstimateS v.9.1.0^25^. A rarefaction curve depicts new species accumulated (y-axis) against number of samples or individuals collected (x-axis). Smoothed curves with 95% confidence intervals representing the statistical expectation of species accumulation are generated through iterative resampling of the species by sample matrix. Such curves generally grow rapidly at first as most common species are collected, and soon plateau as only the rarest species are collected in successive collections. Sample based rarefaction curve estimates “species density”, i.e., number of species detected per sample, and individual based rarefaction curve estimates “species richness”, i.e., number of species per individual collected. Significant differences in diversity were determined based on lack of overlap between the 95% confidence intervals. The total number of species and the number of unique species for each vane color was reported. In addition, the number of shared species for each pairwise comparison of trap colors was calculated. Sorensen (incidence based) similarity indices were generated for each pairwise comparison of vane colors using EstimatesS v 9.1^25^ in order to characterize the extent of species similarity among vane colors.

Redundancy analysis (RDA), a constrained ordination approach was used to examine community assemblage patterns associated with trap color using Canoco v4.5^25^. Vane colors were coded as dummy variables and were used as environmental predictor variables. Because of the preponderance of low abundance species, data were aggregated at genus level. Counts of bees within each bee genera were considered as response variables for each combination of trap color and plot. In the analysis, only genera higher than 1% abundance were included. Square-root transformation was done before analysis and species data were centered for visualization purposes. Significant differences among trap colors were determined using Monte Carlo permutation (n = 499) and stepwise forward selection^26^. Associations among bee genera and trap colors were visualized using biplots drawn using CanoDraw^26^.

## Results

### Wild bee species abundance and richness and association with different vane color types of sampling traps

**Figure 2:**
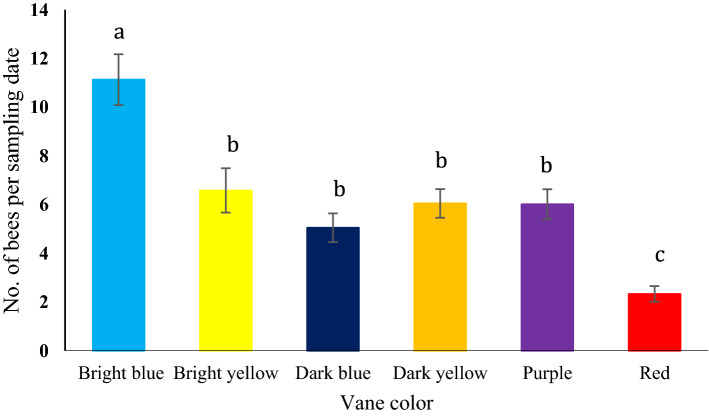
Differences in capture rate (least squares means ± SE) of bees in passive traps with six different colored vanes (bright blue, bright yellow, dark blue, dark yellow, purple, red) in livestock pasture system.

A total of 4416 insect samples were captured in all colored passive vane traps during the entire study period. Among them, 2230 samples were bees belonging to five families, 22 genera, and 49 species. Non-bee species were excluded from further analysis due to resource limitations in identifying and processing those samples. Bee abundance (Table 1; Fig. 2) differed significantly among vane colors (F _(5,15)_ = 9.787; *P* < 0.001). Based on the Bonferroni-correction post hoc comparison test, traps with the bright blue vanes captured the highest number of bees, while the red color vanes captured the least number of bees (*P* < 0.001; Fig. 2). Differences among bright yellow, dark yellow, purple, and dark blue vanes traps were not significantly different in terms of total bee capture rate (Fig. 2).

**Table 1:** Family and species list of bees collected in passive traps with different colored (bright blue, dark blue, dark yellow, bright yellow, purple, and red) vanes. Percent abundance of total species in each trap is presented.

Family	Species	Author	Vane color and Bee species (% of total)					
			Bright blue	Dark blue	Dark yellow	Bright yellow	Purple	Red
Halictidae	*Agapostemon texanus*	Cresson	11.83	9.9	10.47	14.68	6.65	9.29
	*Agapostemon sericeus*	(Forster)	0.15			0.25	0.28	
	*Agapostemon virescens*	(Fabricius)				0.25	0.28	
	*Augochlora pura*	(Say)			0.28	0.25		0.71
	*Augochlorella aurata*	(Smith)	13.17	15.51	45.18	47.59	12.47	30.71
	*Augochloropsis metallica*	(Fabricius)	0.15		2.2	0.25		
	*Halictus ligatus*	Say	4.79	2.97	5.23	4.56	5.54	5
	*Halictus parallelus*	Say	1.8	2.97	3.58	2.28	5.54	5
	*Halictus rubicundus*	(Christ)			0.28			
	*Lasioglossum disparile*	(Cresson)	20.36	11.22	13.77	13.92	29.64	18.57
	*Lasioglossum hitchensi*	Gibbs	0.3		0.28			
	*Lasioglossum imitatum*	(Smith)	15.72	12.21	7.16	4.3	9.7	15.71
	*Lasioglossum pectorale*	(Smith)	2.1	1.32	1.1	0.51	3.05	0.71
	*Lasioglossum tegulare*	(Robertson)	0.15					
	*Lasioglossum lustrans*	(Cockerell)	0.3		0.28	0.76		
	*Halictus tripartitus*	Cockerell	0.75	0.66	0.55	0.51	0.55	2.14
	*Lasioglossum trigeminum*	Gibbs					0.28	
	*Lasioglossum sp.*	Curtis	0.15		0.55	0.25		
	*Nomia nortoni*	Cresson	0.15	0.33				
Apidae	*Apis mellifera*	Linnaeus	0.45	1.32		0.76	0.55	1.43
	*Bombus bimaculatus*	Cresson	0.6					
	*Bombus fervidus*	(Fabricius)	0.75					
	*Bombus griseocollis*	(De Geer)	1.35	0.33	0.83	1.01	3.32	0.71
	*Bombus impatiens*	Cresson	1.2	1.98	1.1	0.51	0.55	2.14
	*Bombus pensylvanicus*	(De Geer)	2.84	0.33	1.93	2.03	0.83	
	*Ceratina calcarata*	Robertson			0.28			0.71
	*Ceratina dupla*	Say				0.25		
	*Ceratina strenua*	Smith	0.3	1.98	0.55	0.25	0.55	
	*Diadasia afflicta*	(Cresson)	0.15					
	*Diadasia enavata*	(Cresson)		0.33				
	*Melissodes bimaculatus*	(Lepeletier)		0.66		0.25	0.28	
	*Melissodes communis*	Cresson	1.2	1.32	0.83		1.94	0.71
	*Melissodes comptoides*	Robertson	0.6	0.99		0.25	0.83	0.71
	*Melissodes denticulatus*	Latreille	0.15	0.99		0.51	0.28	
	*Melissodes sp.*	Robertson	0.15	0.33			0.28	0.71
	*Melissodes vernoniae*	(Say)	9.88	28.05	2.20	1.77	13.85	2.86
	*Melitoma taurea*	Smith	0.15					
	*Peponapis pruinosa*	(Say)	0.3				0.28	
	*Ptilothrix bombiformis*	(Cresson)	5.69	2.97		0.51	1.94	
	*Svastra obliqua*	(Say)	0.15			0.51	0.28	
	*Svastra atripes*	(Cresson)	0.15					
	*Triepeolus lunatus*	(Say)			0.28			
	*Xenoglossa strenua*	(Cresson)	1.35	0.99				0.71
	*Xylocopa virginica*	(Linnaeus)	0.45		0.55	0.51		0.71
Andrenidae	*Calliopsis andreniformis*	Smith				0.25		
Colletidae	*Hylaeus mesillae*	(Cockerell)						0.71
Megachilidae	*Megachile brevis*	Say	0.3		0.28			
	*Megachile montivaga*	Cresson			0.28			
	*Megachile campanulae*	(Robertson)		0.33		0.25	0.28	
	Total species		36	24	25	29	26	20

**Figure 3:**
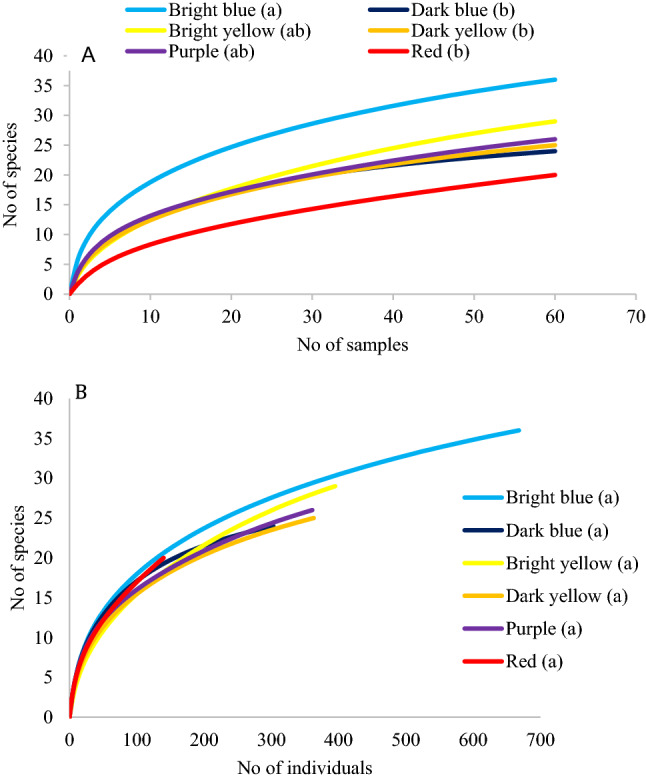
Rarefaction curves showing accumulation of the number of species in relation to the number of samples (**A**) and number of individuals (**B**). Different small letters after color type inside parenthesis indicates significant differences based on non-overlapping 95% confidence intervals.

Sample-based rarefaction curve revealed significantly higher species accumulation in the traps with bright blue vanes compared to dark yellow, dark blue, and red vanes, whereas other trap vane colors (bright yellow and purple) did not differ from any of the other trap colors (Fig. 3A). However, significant differences in species accumulation were not detected among vane colors using individual-based rarefaction (Fig. 3B).

**Figure 4:**
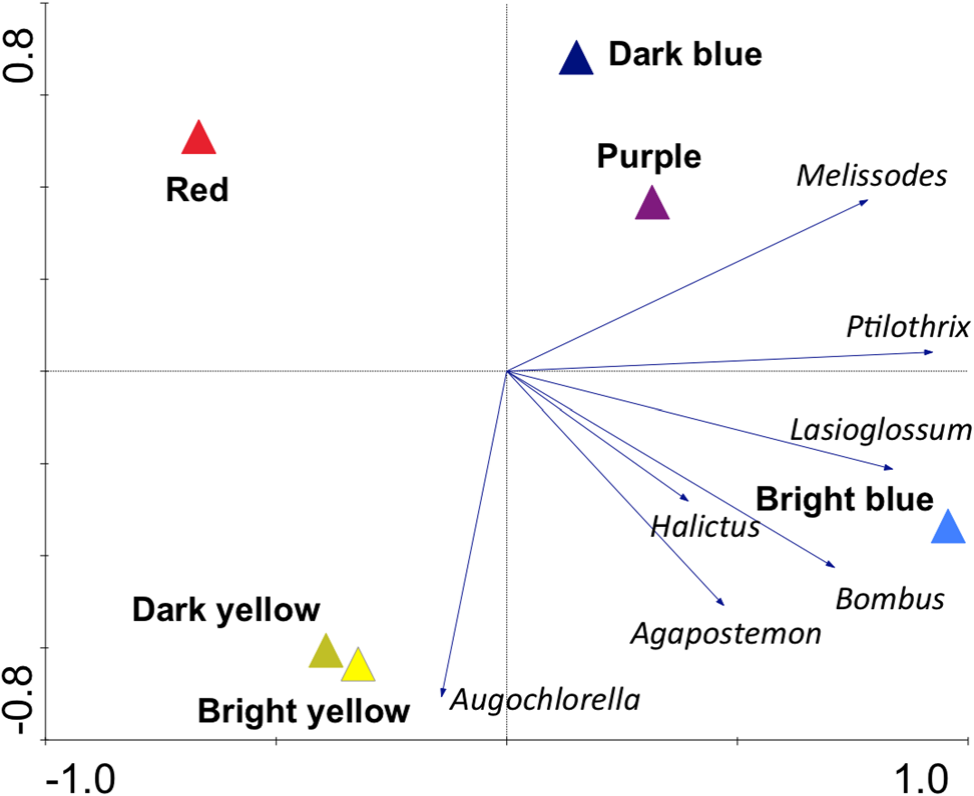
Ordination (redundancy analysis; RDA) biplot showing the association of bee genera and different colored vanes.

Ordination analysis revealed that bee assemblages differed among trap colors (Fig. 4). Stepwise forward selection revealed that bee assemblages in bright blue vane traps (F = 8.62, P = 0.002) differentiated the most from other trap colors, followed by red (F = 4.14, P = 0.012) and dark blue (F = 4.89, P = 0.006). The biplot depicts the first two ordination axes, of which axis 1 explains 41.9% of the species data and Axis 2 explains an additional 21.3% of the species data. Genus vectors on the biplot indicate that bee genera exhibited varying levels of association with trap colors along a gradient from blues to yellows (Fig. 4). *Melissodes* bees were associated with dark blue, purple and bright blue traps. *Ptilothrix, Lasioglossum* and *Bombus* bees showed a stronger association with bright blue traps in particular. *Agapostemon* was associated with both bright blue and bright yellow traps, whereas *Augochlorella* was more closely associated with bright yellow and dark yellow traps. *Halictus* trended toward bright blue, but the short vector suggests a weaker association. No bee genera were strongly associated with red traps.

Most of the bees recorded in this study were from the Halictidae family (77.57%). Of the total bees, 30% were collected from traps with bright blue vanes, 17.71% from bright yellow, 16.27% from dark yellow, 16.18% from purple, 13.58% from dark blue and 6.27% from traps with red vanes. The most abundant species was *Augochlorella aurata* (Smith) (25.78% of total), then *Lasioglossum disparile* (Cresson) (18.29%), *Lasioglossum imitatum* (Smith) (10.85%), *Agapostemon texanus* (Cresson) (10.85%) and *Melissodes vernoniae* (9.865%) and *Halictus ligatus* (Say) (4.708%). Descriptive diversity statistics for each of the trap colors, shows that bee abundance and richness was highest in bright blue traps and followed by bright yellow traps. Abundance and richness were lowest in red traps (Table 2). Bright blue traps also had the greatest number of unique species (Table 2). Shared species and similarity index values based on pairwise comparisons of trap colors depict varying levels of complementarity (i.e. dissimilarity) among different traps colors (Table 2), although these comparisons are limited by the lowest species richness level in any pairwise comparison. The highest levels of dissimilarity were found between purple and dark yellow traps, and between dark blue and dark yellow traps (Table 2). The lowest level of dissimilarity was found between purple and dark blue (Table 2).

**Table 2:** A comparison of bee diversity and similarity measures among six colors of vane traps deployed in Arkansas livestock pastures.

	Vane color					
	Bright blue	Bright yellow	Dark blue	Dark yellow	Purple	Red
*Abundance*	668	395	303	363	361	140
No. species	36	29	24	25	26	20
No. uniques	6	2	1	3	1	1
*Shared species*						
Bright blue	–					
Bright yellow	23	–				
Dark blue	21	19	–			
Dark yellow	20	18	14	–		
Purple	22	22	21	14	–	
Red	17	15	16	15	15	–
*Similarity*^*1*^						
Bright blue	–					
Bright yellow	0.71	–				
Dark blue	0.70	0.72	–			
Dark yellow	0.66	0.67	0.57	–		
Purple	0.71	0.80	0.84	0.55	–	
Red	0.61	0.61	0.73	0.67	0.65	–

^1^Sorensen classic similarity indices.

### Light reflectance properties of vanes of sampling traps

**Figure 5:**
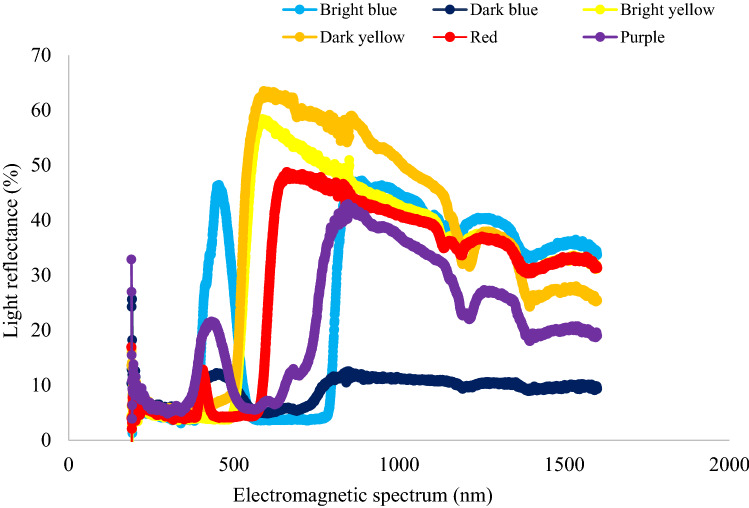
Light reflectance spectrum of different colored vanes of passive traps used for sampling bee communities in pasture system.

**Figure 6:**
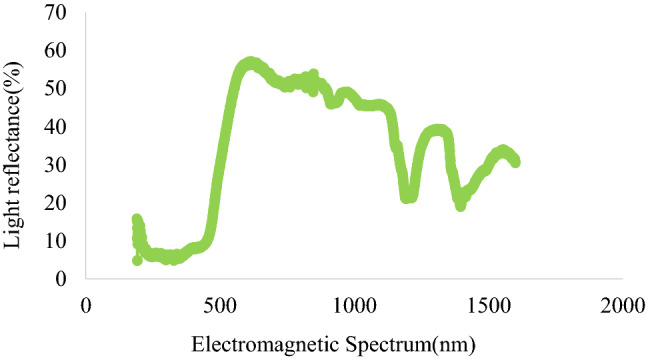
Light reflectance spectrum of the collection jar of the traps used for sampling bees.

Light reflectance spectrum of vanes of all traps used in this study were different from each other (Fig. 5). The same type of collection jar was used for all traps, and the light reflectance curve of the jar showed the reflectance peak at 600 nm (Fig. 6). Bright blue vane had a higher light reflectance that peaked twice in the spectrum, initially at 455 nm within the wavelength ranging from 400–600 nm, and later at 876 nm. In contrast, the light reflectance from the dark blue vane was relatively lower but peaked twice (at 450 nm and 850 nm). Bright yellow vanes had a reflectance peak at 598 nm that gradually decreased with increasing wavelength, and similar pattern was also observed in the case of dark yellow vanes (Fig. 5). Purple vanes showed an initial small peak of light reflectance around 450 nm with range of frequency from 350–500 nm, but later showed higher reflectance that peaked at 879 nm. Similarly, red vanes had a very small initial peak at 390 nm but showed higher light reflectance (~ 48%) later at 661 nm (Fig. 5). Passive traps with colored vanes of higher light reflectance (within 400–600 nm range) attracted the highest number bee species in this study.

## Discussion

This study reveals that various measures of bee diversity-including abundance, richness, and assemblage patterns are influenced by vane color and light reflectance patterns when passively sampling bees with vane traps. In particular, brightly colored vanes with higher light reflectance within 400–600 nm range attracted a greater diversity of bees in traps placed in a livestock pasture ecosystem. Effectiveness of blue and yellow vane traps had been compared previously in different ecosystems, for instance in apple orchards^17^, both woodland and open agriculture farmland^13^, and adjacent to *Helianthus spp*. (Asteraceae) field^27^. In all these studies, blue vane trap captured more bee species and 5–6 times more individuals compared to yellow vane trap.

In the current study, we assessed a different design and size of vanes and a wider array of vane colors and reflectance patterns attached to sample collection jars. In particular, we used bright blue and yellow vanes that were made of plastic sheets covered with a micro-prismatic retro-reflective sheeting that provides better daytime and nighttime brightness as well as high visibility and durability. These vanes showed higher light reflectance and captured the most bees and bee species in this study (Table 2). Similar material was used on red vanes as well, but the light reflectance from those vanes was relatively lower, and as a result captured fewer bees. Traps with bright blue vanes performed especially well in terms of rates of bee capture (Fig. 2; 11.1 bees per trap per sampling date) and rates of species accumulation (Fig. 3). Bright yellow traps exhibited the second highest values for capture rates (Fig. 2; 6.6 bees per trap per sampling date) and species accumulation (Fig. 3), but these rates were not deemed significantly different from some other colors in which the reflective sheeting was not used, such as dark yellow, dark blue and purple.

Bees use visual clues for detection, recognition, and memorization of floral resources in the foraging landscape^7,28^. The intensity of light reflected from different colors of vanes in traps affect number of bees attracted toward the trap^10^. Most bees can recognize colors that fall between 300 to 600 nm visual spectrums^29^. While the information related to the vision of many solitary and wild bees is not available, in the case of honey bees (*Apis mellifera*), color vision is trichromatic with highly sensitive photoreceptors at 344 nm (ultraviolet), 436 nm (blue) and 544 nm (green)^30^.

In this study, colored vanes at a higher light reflectance between 400 to 600 nm attracted the highest number bee species in these passive traps. Capture rate differed among traps with different colored vanes in the current study, which can be explained by sensitivity of visual spectrum of bees and variation in the light reflectance of vanes of these traps. For example, bright blue vanes had two peaks of higher light reflectance, initially in 450–455 nm range and second peak with > 800 nm. Such higher reflectance peak within the optimal range of bee vision may have played an important role in attracting abundant and diverse bee species to these passive traps. Similarly, bright yellow captured second largest number of bees, also had higher light reflectance peak within 600 nm but gradually decreased with increasing wavelength. Though bees have color spectrum from UV to orange^31^, they are sensitive to color spectrum between blue, green and ultraviolet^32^, which is a type of trichromatic vision system^28^. In one study^33^, red color vanes showed relatively lower light reflectance within 600 nm range, but had higher reflectance later in the spectrum, and this could be a reason why a low number of bees were collected in the traps. Past research showed contradictory views regarding the ability of bees to perceive red color. For instance, an early researcher in this field^33^, reported that bees recognize red color objects; however, other researchers had reported inability of bees to perceive^34^ or discriminate red from other colors^35,36^. It was argued that the bees see up to 650 nm in the visual spectrum and may not miss red colored flowers while foraging. However, other factors such as background (vegetation) color could also be contributing to bees’ ability to navigate different vane or flower colors in a livestock pasture landscape. Generally bees use color contrast to locate flower source, and hence neutral colors such as white are usually ignored^29^. Ultraviolet signal can make flowers more or less attractive to bees depending on whether it increases or decreases color contrast^37^. For example, UV color component in yellow^38^ and red^39^ flower increases chromatic contrast of these colored flowers with their background contributing attractiveness to the flowers. However, UV-reflecting white flowers decreases attractiveness for bees^40^.

Different species of bees responded to different colors of vane traps. Out of the 49 bee species collected in this study, only nine bee species were found in all vane color types, whereas 14 species were found in only one trap color. For instance, out of five bumble bee species, two were found in all six vane colors, one was found in five colors, and two species (*Bombus bimaculatus* and *B. fervidus*) were only found in the traps with bright blue vanes. Many of the species that were only found in one trap color- *Calliopsis andreniformis* (1, bright yellow), *Ceratina dupla* (1, bright yellow), *Diadasia afflicta* (1, bright blue), *Diadasia enavata* (1, dark blue), *Halictus rubicundus* (1, dark yellow), *Hylaeus mesillae* (1, red), *Lasioglossum tegulare* (1, bright blue), *Lasioglossum trigeminum* (1, purple), *Megachile montivaga* (1, dark yellow), *Melitoma taurea* (1, bright blue), *Svastra atripes* (1, bright blue), and *Triepeolus lunatus* (1, dark yellow) were singletons and it was impossible to know if this represented a true preference or pattern. Our analysis of assemblage patterns after aggregating bees at the genus level, did show a gradient-like response in bee-color associations (Fig. 4), ranging from dark blue to yellows (with no strong associations found with red vanes). These patterns may be used to guide future (passive trap-based) sampling efforts to monitor bee diversity or to target specific bee species in livestock pastures or other ecosystems. While the bright blue and yellow traps with reflective sheeting were particularly attractive to bees, dark blue and purple traps also had relatively high levels of abundance and richness and collected higher number of *Melissodes*. Purple, as a color, is less commonly used than blue and yellow traps in bee monitoring. While this study shows that purple may be a viable option for bee collection, it’s similar assemblage pattern (Fig. 4) and low level of complementarity with dark blue traps (Table 2) suggests that it may be redundant with blue traps that are already commonly used. Differences in species- and sex-specific associations of bees with different colors of sampling traps had also been reported in previous studies^41^.

Most of the bees collected in the current study were from Halictidae family (77.6%) followed by Apidae. However, few bee species in the families Andrenidae, Colletidae, and Megachilidae were collected. Consistent with our findings, others^42^ reported that bees of the Halictidae family were the most abundant bees in rangeland of Texas. The most common species found in this study were *Au. aurata*, *L. disparile*, *L. imitatum*, and *Ag. texanus*). In our previous studies we have found similar bee diversity in this study region^18^. Pollinator species richness and diversity as well as population distribution in livestock pasture vary during the season^43^. Mid-July to mid-August is the latter half of the summer season in the Southeastern USA, and the sampling period may have missed bee species that emerge earlier in the season and are reported in other studies^42,43^.

Overall, the findings of this study showed that the wild bees responded differently to passive traps with colored vanes of different light wavelength and reflectivity when deployed in a livestock pasture ecosystem. Among six different colors of vanes (dark blue, bright blue, dark yellow, bright yellow, purple and red), the bright blue traps captured the highest number of individuals and species of bees. This could be due to an appropriate match between the visual spectrum of bees and the light reflectance spectrum of vanes, which were made of a micro-prismatic retro-reflective material. Bees responded similarly to traps with other colors of vanes, except for red vane traps, which captured the lowest number of bees. The findings of this study would be useful in understanding bee vision and responses to passive traps, and, such information would help in optimizing bee sampling methods for future monitoring efforts.

## Supplementary Information

Supplementary Information.
