# Supplementary material for: Wild bees respond differently to sampling traps with vanes of different colors and light reflectivity in a livestock pasture ecosystem
Source: Sci Rep. 2022 Jun 13;12:9783. doi: 10.1038/s41598-022-10286-w (PMC9192518; doi:10.1038/s41598-022-10286-w)
Supplement: Supplementary file 1 — Supplementary Information. [file 41598_2022_10286_MOESM1_ESM.docx]

**Supplementary File**

**Table S1.**

Species percentage per seed mixes used for establishing floral resource plantings in study site.

| **1. Tall grass inexpensive seed mix (Prairie Moon, Winona, MN)** | | | | | | |
| --- | --- | --- | --- | --- | --- | --- |
| *Wildflowers* |  |  | |  |  |  |
| *Aster novae-angliae* (1.01%) | |  | |  |  |  |
| *Astragalus Canadensis* (0.51%) | | | |  |  |  |
| *Dalea purpurea* (1.52%) | |  | |  |  |  |
| *Echinacea pallida* (4.54%) | |  | |  |  |  |
| *Echinacea purpurea* (2.53%) | |  | |  |  |  |
| *Eryngium yuccifolium* (2.88%) | | | |  |  |  |
| *Gentiana flavida* (1.01%) | |  | |  |  |  |
| *Heliopsis helianthoides* (1.52%) | | | |  |  |  |
| *Lespedeza capitata* (1.10%) | |  | |  |  |  |
| *Monarda fistulosa* (1.01%) | |  | |  |  |  |
| *Parthenium integrifolium* (2.53%) | | | |  |  |  |
| *Penstemon digitalis* (1.52%) | |  | |  |  |  |
| *Ratibida pinnata* (1.52%) | |  | |  |  |  |
| *Rudbeckia hirta* (4.05%) | |  | |  |  |  |
| *Rudbeckia subtomentosa* (0.51%) | | | |  |  |  |
| *Rudbeckia triloba* (1.52%) | |  | |  |  |  |
| *Silphium integrifolium* (1.10%) | | | |  |  |  |
| *Silphium laciniatum* (1.01%) | |  | |  |  |  |
| *Solidago rigida* (0.51%) | |  | |  |  |  |
| *Zizia aurea* (2.02%) | |  | |  |  |  |
| Total % of wildflowers: 33.92% | | |  |  |  |  |
| *Grasses, sedges and rushes* | |  | |  |  |  |
| *Andropogon gerardii* (22.03%) | | | |  |  |  |
| *Elymus canadensis* (11.01%) | |  | |  |  |  |
| *Panicum virgatum* (1.10%) | |  | |  |  |  |
| *Sorghastrum nutans* (31.94%) | |  | |  |  |  |
| Total percentage by weight for grasses, sedges and rushes: 66.08% | | | | | | |

| **2. Tallgrass Exposed Clay Subsoil Seed Mix (Prairie Moon, Winona, MN)** | | | | | | |
| --- | --- | --- | --- | --- | --- | --- |
| *Wildflowers* |  |  |  |  |  |  |
| *Agastache foeniculum* (1.23%) | | |  |  |  |  |
| *Allium canadense* (1.79%) | |  |  |  |  |  |
| *Aster laevis* (1.35%) | |  |  |  |  |  |
| *Aster novae-angliae* (0.90%) | |  |  |  |  |  |
| *Astragalus canadensis* (0.67%) | | |  |  |  |  |
| *Baptisia alba* (0.90%) | |  |  |  |  |  |
| *Cacalia atriplicifolia* (0.90%) | |  |  |  |  |  |
| *Chamaecrista fasciculata* (14.31%) | | |  |  |  |  |
| *Dalea candida* (1.95%) | |  |  |  |  |  |
| *Dalea purpurea* (2.93%) | |  |  |  |  |  |
| *Echinacea purpurea* (5.38%) | |  |  |  |  |  |
| *Gaura biennis* (2.06%) | |  |  |  |  |  |
| *Heliopsis helianthoides* (1.35%) | | |  |  |  |  |
| *Kuhnia eupatorioides* (1.12%) | |  |  |  |  |  |
| *Lespedeza capitata* (2.24%) | |  |  |  |  |  |
| *Monarda fistulosa* (1.35%) | |  |  |  |  |  |
| *Penstemon digitalis* (1.35%) | |  |  |  |  |  |
| *Pycnanthemum virginianum* (1.35%) | | |  |  |  |  |
| *Ratibida pinnata* (1.35%) | |  |  |  |  |  |
| *Rudbeckia hirta* (3.59%) | |  |  |  |  |  |
| *Rudbeckia subtomentosa* (0.45%) | | |  |  |  |  |
| *Rudbeckia triloba* (0.98%) | |  |  |  |  |  |
| *Silphium laciniatum* (1.35%) | |  |  |  |  |  |
| *Silphium terebinthinaceum* (0.90%) | | |  |  |  |  |
| *Solidago rigida* (0.45%) | |  |  |  |  |  |
| *Verbena hastata* (1.35%) | |  |  |  |  |  |
| *Verbena stricta* (0.90%) | |  |  |  |  |  |
| Total % by wt. wildflowers: 54.45% | | |  |  |  |  |
| *Trees, shrubs and vines* | |  |  |  |  |  |
| *Amorpha canescens* (1.95%) | |  |  |  |  |  |
| Total % by wt. trees, shrubs and vines: 1.95% | | | |  |  |  |
| *Grasses, sedges and rushes* | |  |  |  |  |  |
| *Andropogon gerardii* (9.77%) | | |  |  |  |  |
| *Elymus canadensis* (9.77%) | |  |  |  |  |  |
| *Elymus virginicus* (7.17%) | |  |  |  |  |  |
| *Juncus dudleyi* (0.45%) | |  |  |  |  |  |
| *Panicum virgatum* (0.98%) | |  |  |  |  |  |
| *Sorghastrum nutans* (13.67%) | |  |  |  |  |  |
| *Sphenopholis obtusata* (1.79%) | | |  |  |  |  |
| Total % by wt. grasses, sedges and rushes: 43.60% | | | |  |  |  |

| **3. Buck’s Hangout (Hamilton Native outpost, Elk creek, MO)*** | | | | | |
| --- | --- | --- | --- | --- | --- |
| *Chamaecrista fasciculata* | |  |  |  |  |
| *Chasmanthium latifolium* | |  |  |  |  |
| *Coreopsis lanceolate* | |  |  |  |  |
| *Coreopsis tinctoria* |  |  |  |  |  |
| *Dalea purpurea* |  |  |  |  |  |
| *Desmanthus illinoensis* | |  |  |  |  |
| *Elymus virginicus* |  |  |  |  |  |
| *Heliopsis helianthoides* | |  |  |  |  |
| *Lespedeza virginica* | |  |  |  |  |
| *Penstemon digitalis* |  |  |  |  |  |
| *Ratibida pinnata* |  |  |  |  |  |
| *Rudbeckia hirta* |  |  |  |  |  |
| *Solidago nemoralis* |  |  |  |  |  |
| *Strophostyles leiosperma* | |  |  |  |  |
| *Tridens flavus* |  |  |  |  |  |
| *Total percentage by weight for each species is not available for this seed mix type. | | | | |  |
